# eIF6 links histone acetylation with translational control of HDAC in skeletal muscle

**DOI:** 10.1007/s11010-026-05637-4

**Published:** 2026-07-29

**Authors:** Alessandra Scagliola, Annarita Miluzio, Ivan Ferrari, Daniel Brina, Sara Ricciardi, Stefano Biffo

**Affiliations:** 1 University of Milano, Milan, Italy; 2 Istituto Nazionale Genetica Molecolare “Romeo ed Enrica Invernizzi”, Milan, Italy; 3 Biosciences Department, Università degli Studi di Milano, Milan, Italy

**Keywords:** eIF6, RiboSeq, Protein synthesis regulation, Histone acetylation, Skeletal muscle

## Abstract

Skeletal muscle is crucial for glucose regulation and amino acid storage, significantly influencing overall metabolic balance. Its function is tightly regulated by complex mechanisms, with histone acetylation as a key epigenetic control point. Our previous work identified eIF6 as a key regulator of muscle energy homeostasis and demonstrated its role in modulating histone acetylation in the liver. However, whether similar epigenetic mechanisms underpin eIF6’s effects in muscle remains undetermined. To investigate this, we measured H3K9 acetylation levels and HDAC activity both in vivo, using eIF6^+/–^ mice, and in vitro, following eIF6 depletion. Our findings demonstrate that eIF6 downregulation in C2C12 myoblasts drives an increase in histone acetylation, a pattern also evident in heterozygous eIF6 primary satellite cells, both in their undifferentiated state and following differentiation. In vivo, eIF6^+/−^ mice show pronounced histone hyperacetylation, especially in younger animals, which correlates with a specific decrease in class II HDACs, particularly HDAC4 and HDAC5. This trend is further supported by in vitro data and findings from Drosophila eIF6^+/−^ mutants, both of which exhibit decreased HDAC activity. Importantly, the reduction in HDAC4 and HDAC5 activity appears to result from decreased protein levels, driven by eIF6-dependent translational regulation of their mRNAs. All together, these findings establish a link between mRNA translation and histone acetylation in muscle, underpinning translational control as a master regulator of histone acetylation.

## Introduction

Skeletal muscle is the largest metabolic organ, responsible for up to 30% of daily energy expenditure. It plays a crucial role in glucose uptake and usage, and acts as a reservoir of amino acids, thereby significantly influencing overall the metabolic balance at both the organ and systemic levels [1]. Increased metabolic demands and various disease states disturb muscle homeostasis by impairing energy production, inducing cellular stress, and leading to muscle wasting and fatigue. For example, during prolonged exercise, energy reserves may become depleted, while conditions such as cancer and diabetes can accelerate muscle breakdown, disrupt insulin signaling, and promote the accumulation of metabolic byproducts that trigger inflammation and oxidative stress [2]. The maintenance of proper muscle function and systemic metabolic balance requires complex regulation at multiple levels, from genetic control to signal transduction pathways. Proteins synthesis regulation is particularly prominent in muscle [3]. The activity of translation factors in muscle biology is essential to sustain protein synthesis and muscle growth [4]. The buildup of muscle mass requires activation of protein synthesis downstream of nutrient signaling [5]. On the contrary, it is known since long that decreased protein synthesis is the dominant characteristic of muscle wasting and cachexia [6].

Evidence suggests that acetylation and deacetylation of cellular proteins may be involved in the regulation of muscle mass. At the core of this regulation is the acetyl-CoA, a metabolite derived from amino acids, fatty acids, and glucose. Beyond its role as an energy substrate, acetyl-CoA acts as an essential donor for acetylation reactions, one of the most common modifications of histone lysine residues and other signaling proteins, acting as a key regulator of chromatin structure and signal transduction [7]. Histone acetylation is a dynamic and finely regulated process, controlled by two main enzyme families: histone acetyltransferases (HATs), which add acetyl groups, and histone deacetylases (HDACs), which remove them. The balance between these enzymes is essential for preserving muscle physiology and overall systemic metabolic stability and insulin sensitivity [8, 9].

High throughput studies indicate that translation, given its high energetic cost, is the main regulator of gene expression [10]. In several cell types, mRNA expression is not a predictor of protein expression [11]. Besides, translational control is a powerful regulator of metabolism [12]. As such, translation factors regulate the efficiency of protein synthesis downstream of nutrient signaling pathways such as insulin [13]. In spite of extensive evidence indicating that the control of protein synthesis is essential to muscle fitness, its impact on epigenetic changes such as histone acetylation is unknown.

eIF6 is a translation initiation factor that functions independently of mTOR [14]. Its heterozygous deletion has been shown to limit tumor growth and improve survival in a mouse model of Eµ-Myc lymphoma [15]. eIF6 acts as an anti-association factor by binding to the 60S ribosomal subunit, thereby preventing the formation of inactive 80S complexes. Its activity is further modulated by phosphorylation, stimulated by phorbol esters via PKC activation and in response to insulin [16]. In our previous research, we showed that mice heterozygous for eIF6, which have reduced insulin-dependent translation, maintain normal blood glucose levels but exhibit lower cholesterol and triglyceride levels. This regulation involves eIF6 controlling the translation of adipogenic transcription factors [17]. Interestingly, in an effort to explore potential links between drug action and eIF6 activity, we found that the gene expression profile associated with eIF6 depletion closely resembles that induced by HDAC inhibitors like trichostatin A and MS-275. Supporting this, we observed that downregulating eIF6 leads to histone hyperacetylation in the liver and AML12 cells [17]. Further studies in muscle showed that alterations in eIF6 activity affect the expression of genes involved in critical metabolic pathways, such as oxidative phosphorylation, leading to metabolic changes. These changes were associated with decreased exercise performance in eIF6^+/−^ mice. Furthermore, mass spectrometry-based acetylome analysis of muscle tissue revealed numerous acetylated proteins, reinforcing the idea, initially suggested by liver data, that changes in protein acetylation may be a key mechanism through which eIF6 influences muscle physiology [18].

Here, we demonstrate that eIF6 downregulation in C2C12 myoblasts leads to increased histone acetylation, a pattern also observed in heterozygous eIF6 primary satellite cells, both before and after differentiation. In vivo, eIF6^+/−^ mice exhibit histone hyperacetylation, particularly in younger animals, which correlates with a significant reduction in class II HDACs, HDAC4 and HDAC5. This trend is further supported by in vitro data and is mirrored in Drosophila eIF6^+/−^ mutants, both of which display decreased HDAC activity. Importantly, the reduction in HDAC4 and HDAC5 activity appears to result from lower protein levels, driven by eIF6-dependent translational regulation of their mRNAs. Collectively, these findings establish a link among eIF6, translational control and histone acetylation in muscle tissue.

## Results

### eIF6 modulates histones acetylation in vitro, in satellite cells and C2C12 myoblasts

**Fig. 1:**
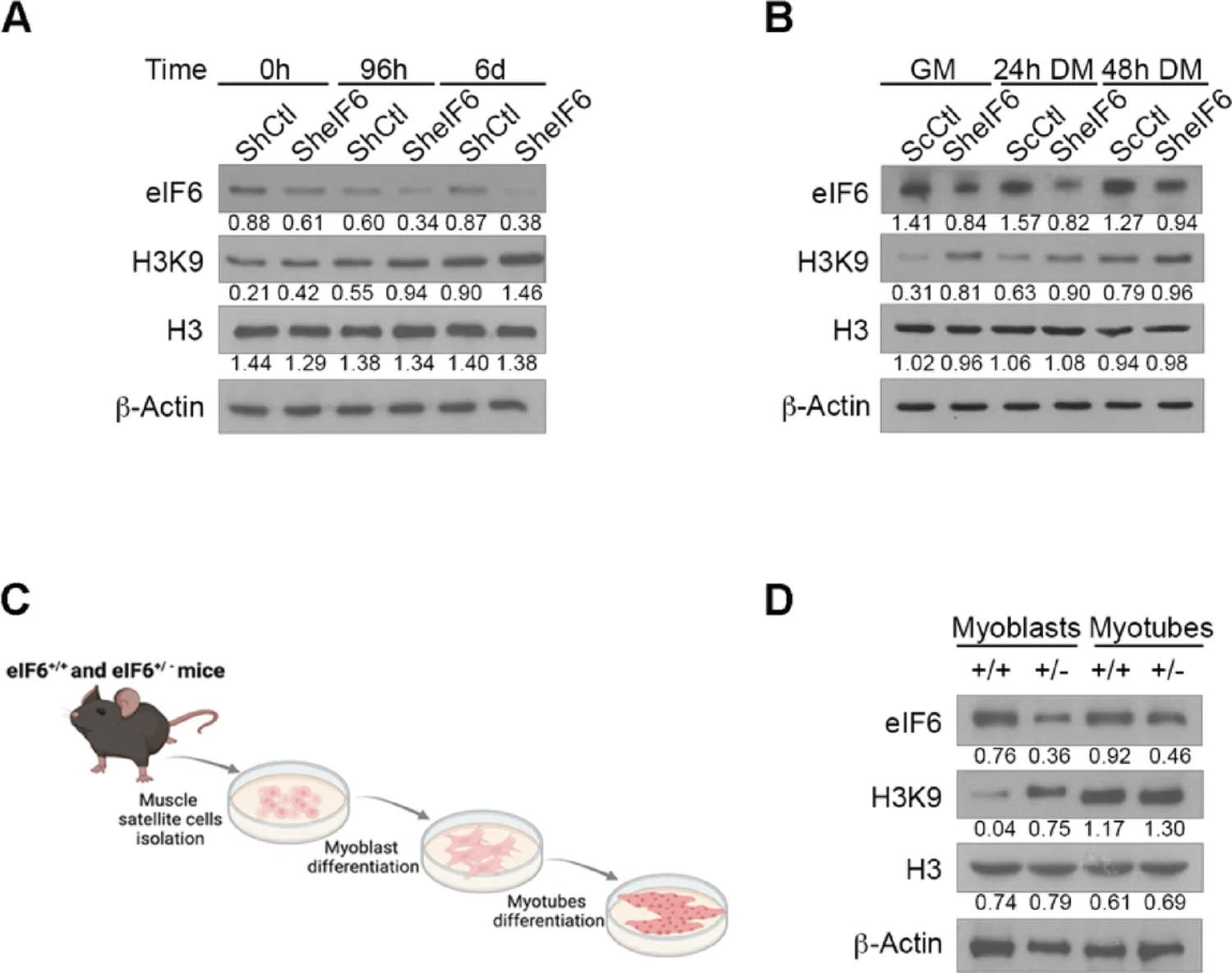
eIF6 modulates histone acetylation in multiple in vitro models and in vivo. **A** Representative immunoblot of two independent experiments in C2C12 cells, illustrating the effects of acute eIF6 downregulation on H3K9Ac levels. After 6 days of shRNA treatment, eIF6 protein levels were significantly reduced, accompanied by a marked increase in H3K9Ac levels. β-Actin was used as loading control. **B** Representative immunoblot of two independent experiments showing H3K9Ac, H3 and eIF6 protein levels in wild-type (WT) and eIF6-downregulated C2C12 cells after 6 days of knockdown, cultured in either proliferating growth media or differentiating conditions. Compared to WT, EIF6-downregulated cells showed higher H3K9Ac in the undifferentiated state, with levels remaining elevated and peaking at 48 h in DM. β-Actin was used as loading control. **C** Overview of the workflow for the differentiation of satellite cells, isolated from the extensor digitorum longus (EDL) muscles of 1-month-old eIF6^+/−^ mice and age-matched eIF6^+/+^ controls. **D** Representative immunoblot of two independent experiments showing H3K9Ac, H3 and eIF6 protein levels in myoblasts and in myotubes from wild-type (WT) and eIF66^+/−^ mice. β-Actin was used as loading control

Initially, we investigated whether eIF6 could affect histone acetylation by acutely reducing its expression in C2C12 cells. After 96 h of shRNA treatment, eIF6 levels were significantly decreased, which was accompanied by an increase in H3K9 Acetylation (H3K9Ac). This increase was most pronounced at 6 days, when eIF6 levels decreased accordingly (Fig. 1A). Therefore, the 6-day time point was chosen for subsequent experiments. Of note, histone acetylation was barely detectable in quiescent C2C12 myoblasts (time 0), with no significant difference in H3K9Ac levels between sh-eIF6 and sh-Ctl cells at this stage (Fig. 1A). We then assessed H3K9Ac level both in sh-Ctl and eIF6-downregulated C2C12 cells after 6 days of knockdown, either in proliferating growth media (GM) or differentiating conditions (DM) (Fig. 1B). During differentiation, WT cells showed a significant increase in H3K9Ac, consistent with normal muscle development (Fig. 1B). In contrast, eIF6-downregulated cells already had higher levels of H3K9Ac in the undifferentiated state, and remained markedly higher during differentiation, peaking at 48 h in DM (Fig. 1B).

Satellite cells (SCs), the stem cells of skeletal muscle, are essential for postnatal muscle growth and tissue regeneration. To form new myofibers, SCs go through several stages, each marked by distinct gene expression patterns. These stage-specific programs are tightly regulated by epigenetic mechanisms that control the accessibility of transcriptional machinery to key gene loci, ensuring the precise and timely expression of myogenic genes. Satellite cells maintain an open, accessible chromatin structure that keeps them ready for activation and differentiation in response to external cues. When differentiation is triggered, these cells generally show increased acetylation of histones H3 and H4, promoting the gene expression necessary for muscle development. We therefore measured H3K9 acetylation (H3K9Ac) level in myoblasts and myotubes, derived from satellite cells, isolated from wild-type (WT) and eIF6^+/−^ mice (Fig. 1C). Western blot analysis confirmed a significant reduction in eIF6 expression in eIF6^+/^^−^ cells compared to controls (Fig. 1D). Western blot analysis further showed that, in WT cells, H3K9Ac levels increased significantly during differentiation, in line with normal muscle development (Fig. 1D). In contrast, eIF6^+/-^ myoblasts displayed elevated H3K9Ac even in the undifferentiated state, and levels remained markedly higher after differentiation, with respect to controls (Fig. 1D). These results demonstrate that eIF6 modulates histones acetylation in vitro, both in satellite cells and C2C12 myoblasts.

### eIF6 modulates histones acetylation in vivo

**Fig. 2:**
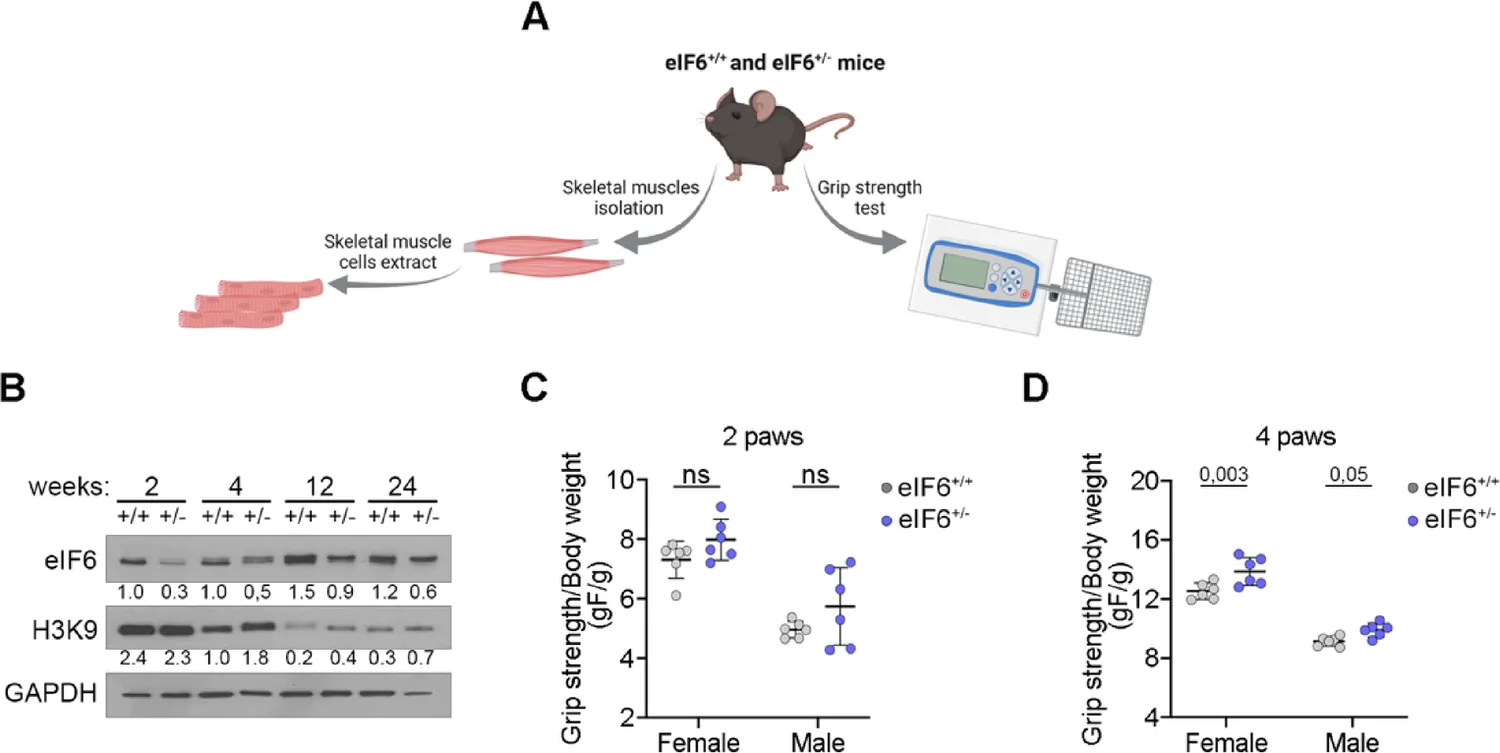
eIF6 levels affect acetylation status and strength of skeletal muscle in vivo. **A** Overview of the workflow for skeletal muscle analysis and force measurement. **B** Representative immunoblot of two independent experiments showing H3K9Ac levels in the TA muscle of WT and eIF6^+/−^ mice at four different ages across their lifespan. eIF6^+/−^ mice maintained higher acetylation levels than WT at all stages, particularly in younger animals. GAPDH was used as loading control. **C**, **D** Grip strength was assessed using a grid-based assay, measuring maximal force in both hindlimbs (**C**) and all four limbs (**D**), in female and male mice (*n* = 6 per group). Values are shown as grip strength normalized to body weight (Fg/grams). Mann-Whitney test: P values are reported in the figure as exact values; ns, not significant

Next, we sought to establish whether the histone acetylation changes seen in vitro with eIF6 depletion also occur in vivo by measuring H3K9Ac levels in the tibialis anterior (TA) muscle of WT and eIF6^+/^^−^ mice across four ages (Fig. 2A). We confirmed that eIF6 protein is reduced in heterozygous mice across all examined developmental stages (Fig. 2B). The TA muscle undergoes significant age-related changes, including decreased mass, lower satellite cell density, increased fibrosis, and metabolic shifts, all of which contribute to the decline in muscle function with age. Since H3K9Ac is a marker of active chromatin, we expected its levels would decrease as the mice aged, making TA an ideal system for tracking epigenetic changes over time. Consistent with this, WT mice showed a significant reduction in H3K9Ac at 24 versus 2 weeks. Although eIF6^+/^^−^ mice also exhibited a decline, they retained higher H3K9 acetylation than WT at every time point examined (Fig. 2B).

Recent studies have demonstrated that increased Smad3 acetylation, via class II HDAC inhibition, enhances muscle strength in dystrophin-deficient mice [19]. Guided by these findings, we evaluated muscle strength in WT and eIF6^+/^^−^ mice. Grip strength test was assessed to measure maximal hindlimb force, normalized to body weight, in both male and female mice (Fig. 2A). Six-weeks-old heterozygote mice showed higher two-paw grip strength compared to WT controls (Fig. 2C), and this advantage was further amplified when all four paws were measured (Fig. 2D), reflecting a gain in muscle strength.

All together, these results agree and support the in vitro data and conclusively demonstrate that eIF6 modulates histone acetylation, suggesting that epigenetic regulation may be a key pathway through which eIF6 impacts muscle physiology.

### eIF6 depletion results in reduced activity of HDAC4 and HDAC5, which correlates with increased histone hyperacetylation

**Fig. 3:**
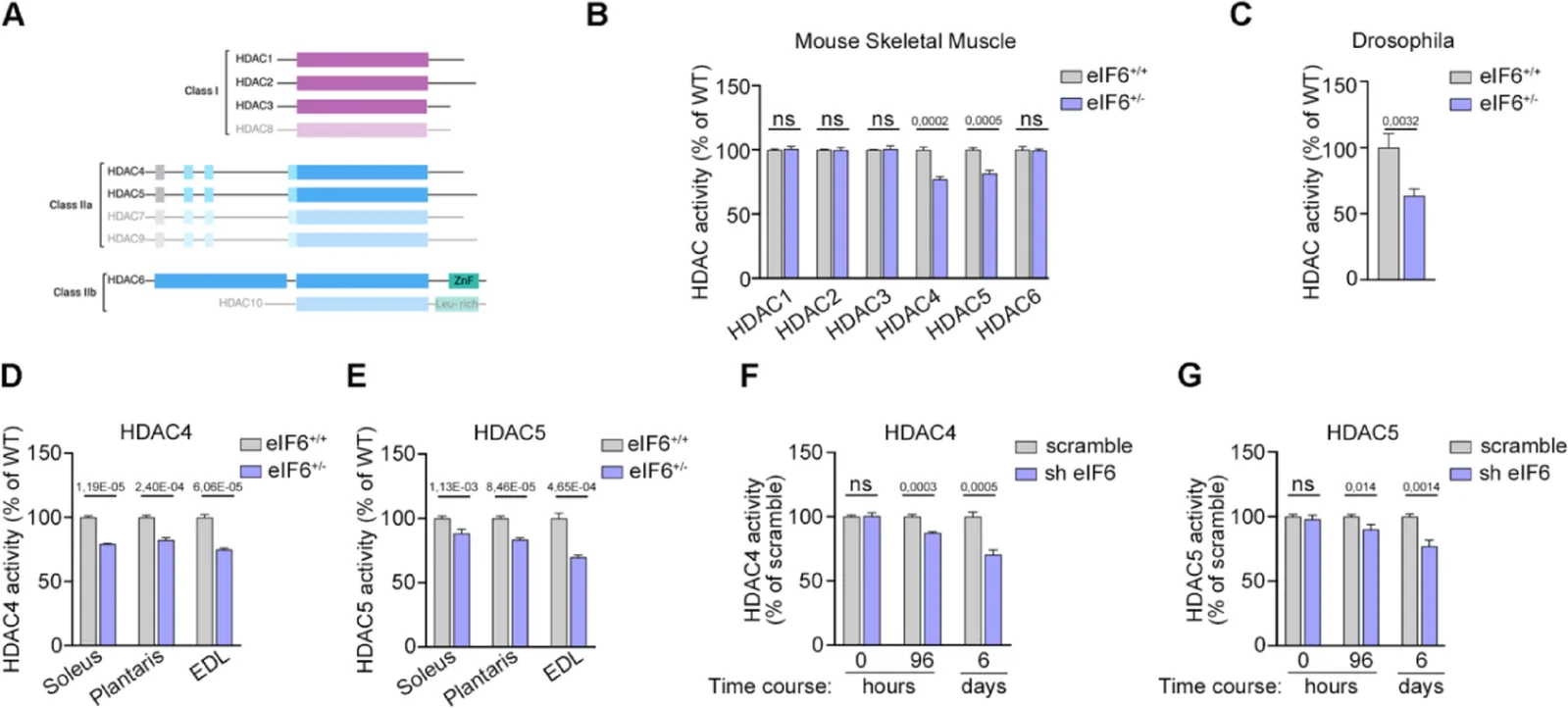
eIF6 depletion leads to decreased activity of HDAC4 and HDAC5, which correlates with histone hyperacetylation. **A** Schematic depiction of the different isoforms of HDAC families. Bars depict the length of the protein. **B** Representative deacetylase activity assay of two independent experiments in tibialis anterior (TA) muscles of 1-month-old WT and eIF6^+/−^ mice showing a specific reduction in class II HDAC activity, particularly HDAC4 and HDAC5, in eIF6^+/−^ mice. Student’s t-test: P values are reported in the figure as exact values; ns, not significant. **C** Global HDAC activity was measured in brains from eIF6^+/−^ Drosophila and their respective controls (*n* = 30 per group). Student’s t-test: P values are reported in the figure as exact values; ns, not significant. **D**, **E** Representative HDAC4 and HDAC5 deacetylase activity assay from two independent experiments in the SOL, PLA, and EDL muscles from WT and eIF6^+/−^ mice. Student’s t-test: P values are reported in the figure as exact values; ns, not significant. **F**, **G** Representative HDAC4 and HDAC5 deacetylase activity assays from two independent experiments, showing reduced activity in C2C12 cells following eIF6 knockdown. Student’s t-test: P values are reported in the figure as exact values; ns, not significant

HDACs are well known for their role in deacetylating lysine residues on histone H3 (Fig. 3A). To identify which specific deacetylases regulate H3K9Ac levels under eIF6 depletion, we evaluated HDAC activity both in vivo, in eIF66^+/−^ mice muscles, and in vitro, in C2C12 cells with eIF6 knockdown, employing a fluorometric assay. Remarkably, analysis of HDAC activity of the tibialis anterior (TA) muscles from 1-month-old WT and eIF6^+/−^ mice revealed a specific decrease in class II HDACs, particularly HDAC4 and HDAC5 in eIF6^+/−^ mice (Fig. 3B). This finding was further supported by data from Drosophila eIF6^+/−^ mutants, which also showed similar reductions in HDAC activity (Fig. 3C).

Given the distinctive contractile and metabolic profiles of muscle fiber types, we examined whether HDAC4 and HDAC5 activity varies among these fibers in eIF6^+/−^ mice. The soleus (SOL), primarily composed of slow, oxidative fibers, and the plantaris (PLA) and extensor digitorum longus (EDL), mainly fast, glycolytic fibers, were analyzed (Fig. 3D, E). Interestingly, HDAC4 and HDAC5 activity was similarly reduced in both the slow, oxidative fibers of eIF6^+/−^ mice and the fast fibers (Fig. 3D, E), despite class II HDACs being predominantly expressed in fast-twitch muscles like PLA and EDL, with lower expression in slow-twitch fibers such as the soleus. We further investigated whether HDAC4 and HDAC5 activity decreases in vitro in C2C12 cells following eIF6 knockdown. After 96 h of shRNA treatment, eIF6 levels dropped significantly (Fig. 1A), leading to a corresponding decline in HDAC4 and HDAC5 activity. This effect was most pronounced at six days, when eIF6 expression was at its lowest (Fig. 3F, G). Together, these findings demonstrate that eIF6 influences histone acetylation by lowering HDAC4 and HDAC5 activity across multiple models, highlighting its role in the epigenetic regulation within muscle tissue.

### The decrease in class II HDAC activity upon eIF6 depletion is due to a reduction in their protein levels

**Fig. 4:**
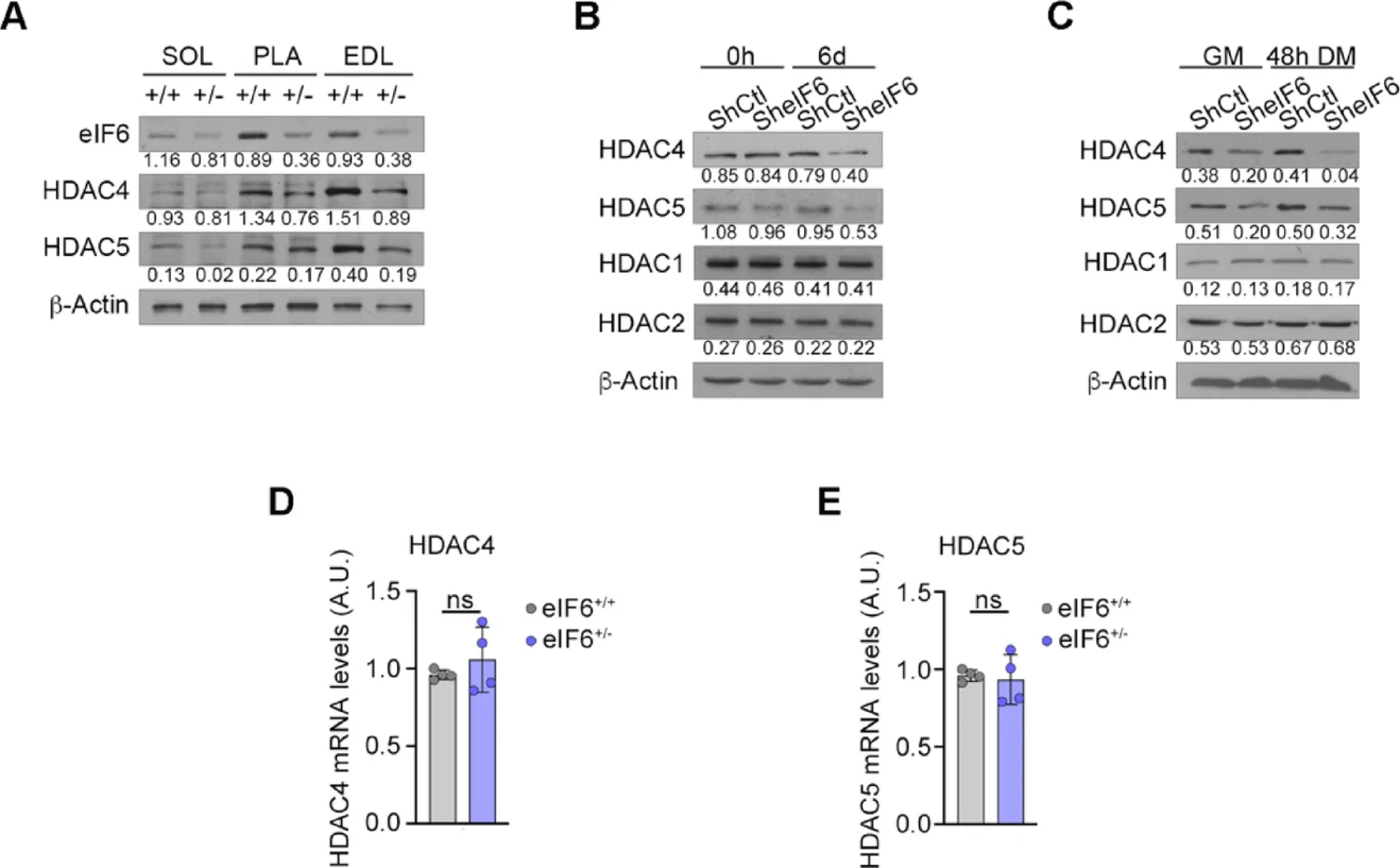
The impairment in Class II HDAC activity upon eIF6 depletion is mainly due to decreased protein abundance. **A** Representative immunoblot of two independent experiments in SOL, PLA and EDL muscles isolated from wild-type (WT) and eIF6^+/−^ mice showing eIF6, HDAC4 and HDAC5 protein levels. β-Actin was used as loading control. **B**, **C** Representative immunoblots of two independent experiments of WT and eIF6-depleted C2C12 cells, before and during differentiation showing decreased HDAC4 and HDAC5 protein levels. β-Actin was used as loading control. **D**, **E** Representaive qRT-PCR of two independent experiments of HDAC4 and HDAC5 transcripts in the TA muscle of WT and eIF6^+/−^ mice showing no significant differences in their mRNA expression levels between the two groups. Values were normalized to GAPDH mRNA. Student’s t-test: ns, not significant

We hypothesized that the reduced class II HDAC activity observed with eIF6 depletion is primarily due to a decrease in the protein levels of these deacetylases. To investigate this, we analyzed HDAC protein expression both in vivo and in vitro. In vivo, we examined the Soleus (SOL), Plantaris (PLA), and Extensor Digitorum Longus (EDL) muscles from the hind limbs of WT and eIF6^+/−^ mice at 1 month of age (Fig. 4A). In vitro, we assessed HDAC levels in WT and eIF6-downregulated C2C12 cells after six days of knockdown, both before and during differentiation (48 h in differentiation media, DM). Immunoblot analyses confirmed that eIF6 depletion in both settings leads to a decrease in HDAC4 and HDAC5 protein levels (Fig. 4A–C). Notably, the greater reduction of class II HDACs in the PLA and EDL muscles of eIF6^+/−^ mice, compared to the relatively smaller decline in the soleus, likely reflects the expression pattern of these HDACs, which are predominantly present in fast-twitch, muscle-dominant fibers like PLA and EDL, with lower expression in slow-twitch, oxidative fibers such as the soleus. Furthermore, quantitative RT-PCR revealed no significant difference in the transcript levels of HDAC4 and HDAC5 between eIF6^+/−^ mice and controls (Fig. 4D, E). Taken together, these results demonstrate that histone acetylation is reduced in skeletal muscle through eIF6-mediated translational, but not transcriptional, regulation of HDAC4 and HDAC5

### Ribo-seq analysis confirms translational control of selected HDACs

**Fig. 5:**
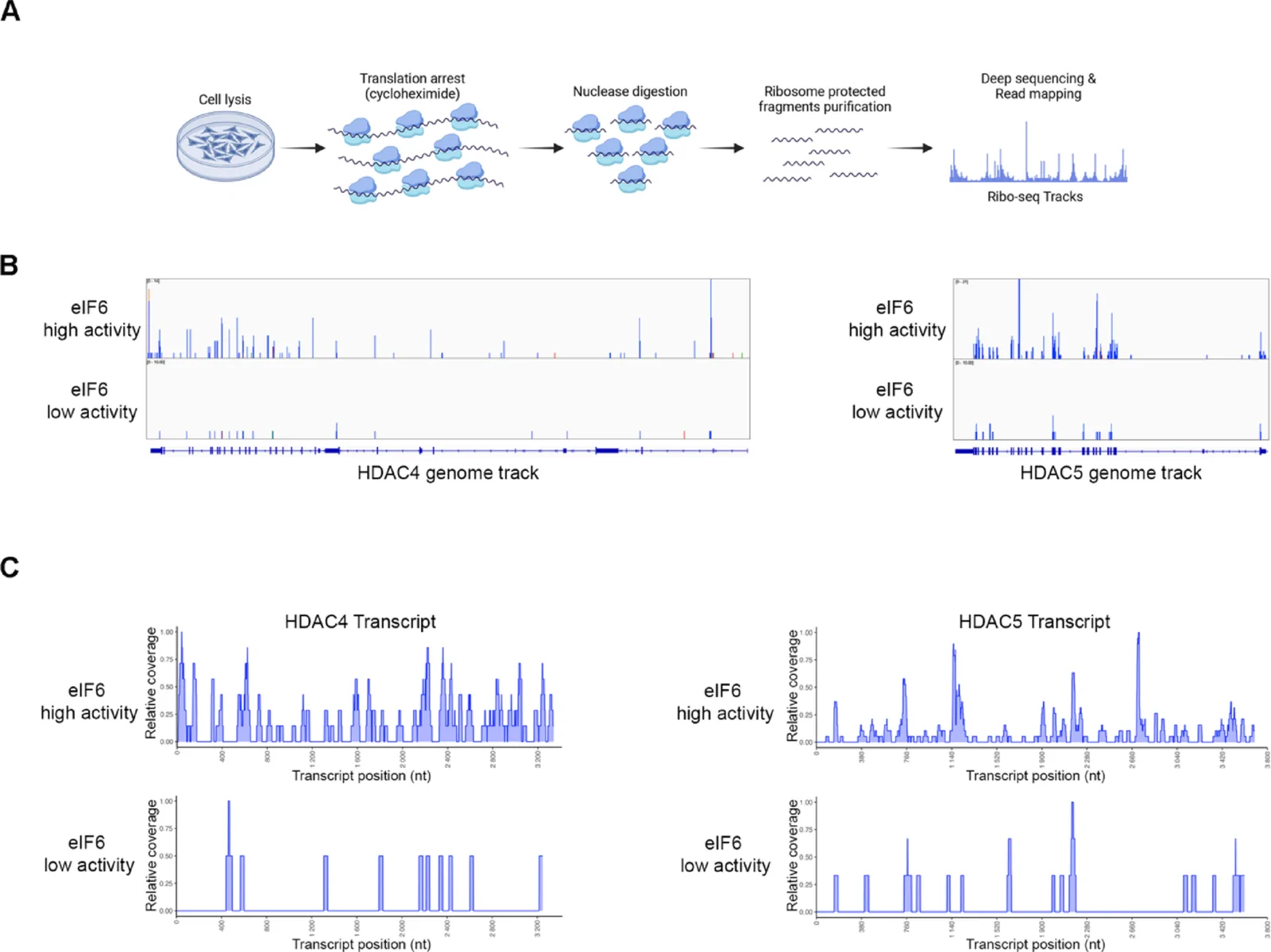
HDAC4 and HDAC5 mRNAs are translationally dependent on eIF6 activity. **A** Scheme of the experimental design to analyze ribosome protected fragments. **B** Genome tracks of HDAC4 and HDAC5 under normal and reduced eIF6 activity. eIF6 low activity refers to the condition where eIF6 activity is reduced to around 30% of maximal levels. **C** Distribution of ribosome footprints along HDAC4 and HDAC5 transcripts, indicating reduced coverage under conditions of low eIF6 activity

Ribo-seq analysis further support translational control of HDAC4 and HDAC5. We have generated a mouse model in which lack of eIF6 phosphorylation results in a drop in the efficiency of mRNA translation due to the impaired recycling at stop codons. The model underlines the efficiency of eIF6 regulated mRNA translation at the single gene level [20]. In this model, ribosome footprint coverage across the HDAC4 and HDAC5 transcripts was markedly reduced (Fig. 5A–C) [21]. These Ribo-seq results align with our protein and mRNA measurements: HDAC4 and HDAC5 protein levels fall in eIF6-deficient muscle and C2C12 cells, while their mRNA levels remain unchanged (Fig. 4A–E). Together, the data indicate that eIF6 acts post-transcriptionally to sustain HDAC4/5 protein synthesis, and that reduced eIF6 activity directly limits ribosome engagement of these transcripts, leading to lower HDAC protein abundance and consequent changes in histone acetylation in skeletal muscle.

## Discussion

Here we report that downregulation of eIF6 results in increased level of H3K9 acetylation. Our investigation started from the serendipitous observation that tissues from heterozygous mice for eIF6 had a transcriptional profile resembling the one of cells treated with common histone deacetylase inhibitors [17]. This finding was accompanied by the confirmation that histone acetylation is increased in tissues exhibiting reduced eIF6 expression. Acetylation is sensitive to acetyl-coA levels. We could rule out the presence of increased amounts of acetyl-coA due to decreased translation, because models in which eIF6 activity and translation are strongly reduced do not show an increase in acetyl-coA levels [20].As eIF6 is a rate-limiting factor for translational regulation [14, 15], the obvious culprit for the effects observed is represented by translational regulation. We found reduced protein levels of two histone deacetylase HDAC4 [22], and HDAC5 [23]. We have additional evidence that other deacetylases could be regulated at the translational level, including HDAC3. However, sirtuin1 [24] levels did not seem affected by eIF6. These data indicate that histone acetylation may be controlled also at the translational level. However, whether additional mechanisms account for increased acetylation is unknown. Globally, our findings raise the important issue of the interaction between the translational machinery and epigenetic modifications, an area so far not explored.

Our study suggests that eIF6 regulates histone acetylation partly via control of HDAC4/5 protein levels. eIF6 depletion reduces HDAC4/5 abundance, leading to decreased deacetylase activity and consequently increased H3K9 acetylation. This mechanism appears to function across different muscle fiber types and may be conserved from Drosophila to mammals. HDAC4/5 are key regulators of muscle metabolism and fiber type specification [25]. Their reduction would be expected to promote oxidative metabolism and slow fiber characteristics, similar to endurance training adaptations. This well aligns with previous observations that chronic eIF6 inhibition increases oxidative phosphorylation, in vivo [26]. However, a previous study also indicated that the whole pattern of acetylation changed in the muscle of eIF6 het mice, including an increase in several mitochondrial proteins, but a decrease in global acetylation. Histones were not analyzed in that study [18]. We will reconcile these findings and offer a simple explanation. Translational regulation of different players of protein acetylation and deacetylation affects multiple proteins that unequivocally lead to a net increase in histone H3K9 acetylation. Overall, eIF6 activity is a master regulator of metabolic downstream events that include mitochondrial efficiency and lipid synthesis [12] and acts as a promitotic factor [14]. In presence of reduced activity of eIF6, the general trend is to delay the progressive loss of histone acetylation that correlates with the establishment of tissue-specific gene expression programs. In muscle cells, H3K9 acetylation shows a striking reduction during myogenic differentiation [27]. Possibly, in conditions of reduced protein synthesis the developmental clock must be slowed down. This speculation is supported by the observation that mutants of translation factors delay, in general and throughout evolution, aging [28].

We should note that aging mostly affect fast-twitch muscles, but we found that HDAC4 activity was reduced in both the slow, oxidative fibers of Eif6+/− mice and the fast fibers, despite class II HDACs being predominantly expressed in fast-twitch muscles like PLA and EDL. Future work should establish the differential impact of the loss of eIF6 in slow and fast fibers, and in aging mice, older than two years.

The gap in knowledge between the association of the translational machinery with epigenetics events is intriguing. As expected, several contributions indicate that epigenetic changes are involved in the regulation of translation factors [29–31]. However, given the fact that ribosomes are an essential, massive component of the cellular machinery, a close analysis of data indicates that the impact of epigenetic changes in the expression of ribosomal proteins or initiation factors is generally minor, if compared to other classes of developmentally regulated proteins. However, the situation may be different if comparing cells in highly divergent situations such as quiescent cells versus their proliferating counterparts. The most compelling cases of crosstalks between epigenetic modifications and the translational machinery are represented by epigenetic modifications of the mRNA that can have a clear effect on the translation of specific mRNAs [32]. However, our study suggests that also epigenetic drivers may be under the control of the translational machinery. Biochemical and genetic data indicate that both eIF6 and eIF4E are rate limiting translation factors that regulate the rate of protein synthesis and the efficiency of specific mRNAs [33, 34]. Thus, the impact of translation factors such as eIF6 or eIF4E on the epigenome deserves a closer look.

## Materials and methods

### Animal model

eIF6^+/+^ and eIF6^+/−^ mice were generated [14] and used in this study, including both males and females. Experiments were carried out on mice from 2 to 24 weeks old. Mice were genotyped and randomly assigned to experimental groups, with no specific inclusion or exclusion criteria. All studies were performed on age-matched animals, comparative analyses were performed in blind and primary cells were always derived from littermates of the same genotype. All experiments involving animals were performed in accordance with Italian national regulations and covered by experimental protocols reviewed by local Institutional Animal Care and Use Committees (IACUC 1088). The Drosophila model used in this analysis has been characterized in [35].

### Culture and differentiation of C2C12 cells

C2C12 mouse skeletal muscle cells were kindly provided by Dr. Davide Gabellini and were originally obtained from the American Type Culture Collection (ATCC, LGC Standards, Sesto San Giovanni, MI, Italy). The C2C12 myoblasts were cultured in growth medium (GM) consisting of Dulbecco’s Modified Eagle Medium (DMEM) (Thermo Fisher Scientific), supplemented with 10% fetal bovine serum (FBS) and penicillin/streptomycin (Invitrogen), and maintained at 37 °C in a humidified atmosphere containing 5% CO₂. Myogenic differentiation was initiated once the cells reached confluence by replacing the culture medium with differentiation medium (DM), composed of the same base medium supplemented with 2% horse serum instead of FBS. The medium was refreshed every two days, and when the desired differentiation stage was reached, cells were collected by centrifugation at 3000 × g for 5 min for subsequent Western blot analysis.

### Lentiviral infection of C2C12 cells

For shRNA experiments, C2C12 cells were infected with lentiviruses carrying either a scrambled shRNA or eIF6 shRNA. The mature antisense sequence of the eIF6 shRNA is: 5′-AGCTTCCTACTAGCACCTG-3′ (Open Biosystems). Lentiviruses encoding either the scrambled shRNA or the eIF6 shRNA were obtained as previously described [17]. Briefly, HEK293T cells were seeded at 125,000 cells/cm² in DMEM supplemented with GlutaMax (Gibco), 10% FBS (Gibco), and 1× penicillin-streptomycin (Thermo Fisher Scientific). The following day, once cells reached approximately 90% confluence, they were transfected with plasmids encoding VSV-G, PMDL g/pRRE, pREV, and the respective shRNAs using calcium phosphate transfection. After 24 h, the medium was replaced with fresh culture medium. Lentiviral particles were collected after 48 h, pelleted by centrifugation at 20,000 × g for 120 min, resuspended in PBS, aliquoted, and stored at −80 °C.

### Satellite cells isolation and culture

Satellite cells were isolated from the extensor digitorum longus (EDL) muscles of 1-month-old eIF6^+/−^ and age-matched eIF6^+/+^ controls, following established protocols [36]. In brief, the EDL muscles were carefully dissected from tendon to tendon and digested in 2% collagenase type I (Sigma) dissolved in DMEM at 35 °C for 70 min. After digestion, the muscles were washed multiple times to remove debris and separate muscle fibers from contaminating cells.

The isolated satellite cells were cultured in growth medium (GM) containing Ham’s F10 supplemented with 20% horse serum, 1% penicillin/streptomycin, 1% glutamine, and 5 ng/ml bFGF (Invitrogen). The cells were maintained in GM for four days, with fresh bFGF added daily, and then collected for subsequent Western blot analysis. To induce myotube formation, cells were plated on wells pre-coated with 10 µg/ml laminin. They were cultured in GM with bFGF for four days, after which the medium was replaced with differentiation medium (DM) consisting of DMEM supplemented with 10% horse serum, 10% fetal bovine serum, 1% glutamine, 1% sodium pyruvate, 50 ng/ml gentamicin, and 0.5% chick embryo extract. Cells were cultured in DM for an additional three days to allow myotube formation, then collected for subsequent Western blot analysis.

### Grip strength test

Muscle strength was assessed for each animal using a Grip Strength Meter (Chatillon DFE II, Columbus Instruments) in both fore paws and all paw modes. Each mouse was gently positioned near the probe until it established a secure grip, then pulled horizon tally at a constant speed of ~ 2.5 cm/s until it released the bar. The maximal peak force (Fg) was recorded. Each mouse was measured three times consecutively with two and four paws, with 15-second intervals between trials. All measurements were performed by the same investigator to minimize variability, and the testing order was randomized.

### SDS-PAGE and western blotting

SDS-PAGE and Western blotting were performed on protein extracts obtained from C2C12 cells, satellite cells, or muscle tissue, following previously described protocols [17, 37]. The primary antibodies used included: mouse polyclonal anti-β-actin (1:4000, Cell Signaling), mouse monoclonal anti-HDAC1 (1:1000, Cell Signaling), mouse monoclonal anti-HDAC2 (1:1000, Cell Signaling), mouse monoclonal anti-HDAC3 (1:1000, Cell Signaling), rabbit polyclonal anti-HDAC4 (1:1000, Cell Signaling), rabbit polyclonal anti-HDAC5 (1:1000, Cell Signaling), rabbit polyclonal anti-HDAC6 (1:1000, Cell Signaling), rabbit polyclonal anti-Acetyl-Histone H3 (1:1000, Cell Signaling), rabbit polyclonal anti- Histone H3 (1:1000, Cell Signaling).

Primary antibody detection was carried out using peroxidase-conjugated secondary antibodies (Amersham, Piscataway, NJ, USA), and signal development was achieved with the SuperSignal West Pico PLUS chemiluminescent substrate (Thermo Fisher Scientific).

### RNA isolation and qPCR

Total RNA was isolated using TRIzol™ Reagent (Thermo Fisher Scientific) following the manufacturer’s instructions. Reverse transcription was performed with SuperScript IV VILO Master Mix with ezDNase™ (Thermo Fisher Scientific). Quantitative PCR (qPCR) was carried out using Platinum SYBR Green qPCR SuperMix-UDG with ROX (Thermo Fisher Scientific) on a QuantStudio 3 Real-Time PCR System (Thermo Fisher Scientific). The primers used were as follows: HDAC4 (forward: 5′-CAGATGGACTTTCTGGCCG-3′; reverse: 5′-CTTGAGCTGCTGCAGCTTC-3′) and HDAC5 (forward: 5′-GAAGCACCTCAAGCAGCAGCAGG-3′; reverse: 5′ CACTCTCTTTGCTCTTCTCCTTGTT-3′). Gene expression levels were analyzed and normalized to the housekeeping gene GAPDH. Relative quantification was calculated using the 2 − ΔΔCt method.

### Enzyme assays

HDAC activity was measured in nuclear extracts from C2C12 cells and muscle tissue, and in total extracts from Drosophila brain.

C2C12 cells and muscle were lysed in ice-cold hypotonic buffer (10 mM HEPES pH 7.9, 10 mM KCl, 0.1 mM EDTA, 10 mM DTT, protease inhibitors) and nuclei were pelleted at 1,500 × g for 5 min at 4 °C. Pelleted nuclei were extracted in high-salt buffer (20 mM HEPES pH 7.9, 0.4 M NaCl, 1 mM EDTA, 25% glycerol, protease inhibitors) for 60 min at 4 °C with rotation and clarified at 12,000 × g for 10 min at 4 °C. Protein concentration was determined, and 800 µg of nuclear extract was incubated overnight at 4 °C with anti-HDAC antibodies to immunoprecipitate target enzymes; immunocomplexes were captured with protein G agarose beads for 16 h at 4 °C, washed, and assayed for deacetylase activity using the Histone Deacetylase Assay Kit (Sigma) following the manufacturer’s instructions.

For Drosophila brain, 30 brains were pooled and homogenized in ice-cold lysis buffer (50 mM Tris-HCl pH 8.0, 150 mM NaCl, 1 mM DTT, 0.5% NP-40, protease inhibitors), clarified at 12,000 × g for 10 min at 4 °C; protein concentration measured and 50 µg of total protein per sample was assayed for HDAC activity using the Histone Deacetylase Assay Kit (Sigma) according to the manufacturer’s instructions.

### Ribo-seq data analysis

Libraries were sequenced at 100 M depth on Illumina NextSeq 2000. Adapter sequences from the sequenced libraries were trimmed using Cutadapt. To identify PCR duplicates, UMI sequences were trimmed and inserted into the read IDs using the *extract_umi* function in the UMI-dedup tool. To remove rRNAs, tRNAs, and/or ncRNAs, the reads were aligned to relevant databases using Bowtie2. Reads that passed these initial filtering steps were then aligned to the transcriptome (Gencode Mouse database version 32) using STAR Aligner. PCR duplicates were subsequently discarded using the *dedup* function in the UMI dedup tool. The resulting reads were ribosome-protected fragments (RPFs) of different lengths, reflecting the conformation of the ribosomes at that moment.

Data were then loaded into R with the *bamtolist* function from the RiboWaltz package, which converts aligned BAM files into a transcript-level format suitable for downstream analysis. From these processed lists, we generated transcript coverage profiles, visualising the distribution of ribosome footprints along selected coding sequences. To aid interpretation, we implemented a plotting workflow that produces IGV-like tracks for each sample, showing coverage across individual transcripts and highlighting differences in read density.

### Quantification and statistical analysis

Statistical analyses were performed using GraphPad Prism (Version 8, GraphPad Software). The data are expressed as mean ± sd. or s.e.m. Student’t *t* test was used to compare pairs of data. The number of independent replicates, error bars, *P* values, and statistical tests are reported in the corresponding figure legends. Mann-Whitney test was used for grip test in mice. The following symbols are used in the figure legends for P values: n.s., not significant.

## Data Availability

The Ribo-seq datathat support the findings of this study have been deposited to the EMBL-EBI data repository ArrayExpress: https://www.ebi.ac.uk/fg/annotare. Project accession: E-MTAB-13319.
